# Towards an appropriate African framework for public engagement with human genome editing: a call to synergistic action

**DOI:** 10.12688/wellcomeopenres.18579.2

**Published:** 2023-05-12

**Authors:** Gerald Michael Ssebunnya

**Affiliations:** 1 Padre Pio Medical Centre, Gaborone, Botswana; 2 Africa Institute for Human Dignity, Gaborone, Botswana

**Keywords:** Human genome editing, germline gene editing, ethics, public engagement, governance, African framework

## Abstract

The CRISPR-Cas9 system has revolutionised the biotechnology of human genome editing. Human germline gene editing promises exponential benefits to many in Africa and elsewhere, especially those affected by the highly prevalent monogenic disorders - for which, thanks to CRISPR, a relatively safe heritable radical therapy is a real possibility. Africa evidently presents a unique opportunity for empirical research in human germline gene editing because of its high prevalence of monogenic disorders. Critically, however, germline gene editing has raised serious ethical concerns especially because of the significant risks of inadvertent and intentional misuse of its transgenerational heritability. Calls for due prudence have become even more pronounced in the wake of the 2018 case of He Jiankui’s ‘CRISPR’d babies’. Meanwhile, Africa is seriously lagging in articulating its position on human genome editing. Conspicuously, there has been little to no attempt at comprehensively engaging the African public in discussions on the promises and concerns about human genome editing. Thus, the echoing key question remains as to how Africa should prudently embrace and govern this revolutionary biotechnology. In this article, therefore, I lay the groundwork for the possible development of an appropriate African framework for public engagement with human genome editing and call upon all stakeholders to urgent synergistic action. I particularly highlight the World Health Organisation’s possible leadership role in promptly establishing the requisite expert working group for this urgent need.

## Disclaimer

The views expressed in this article are those of the author(s). Publication in Wellcome Open Research does not imply endorsement by Wellcome.

## Introduction

There is no doubt that the advent of the CRISPR (Clustered Regularly Interspaced Short Palindromic Repeats)-Cas9 system has revolutionised the biotechnology of human genome editing (HGE) ( Locke, 2020). Indeed, human germline gene editing (GGE) promises exponential benefits to many in Africa and elsewhere, especially those affected by the highly prevalent monogenic disorders - for which, thanks to CRISPR, a relatively safe heritable radical therapy is a real possibility ( Kofler & Kraschel, 2018). Human monogenic disorders include sickle cell disease (SCD), which is reportedly the commonest one worldwide ( Piel *et al.*, 2017). Remarkably, approximately 75% of people with SCD are in sub-Saharan Africa, where the incidence is about 250,000 new births per year ( Macharia *et al.*, 2018). Similarly, autosomal recessive oculocutaneous albinism (OCA) is a highly prevalent monogenic disorder in Africa, especially in Southern Africa. Among the Tonga tribe of Zimbabwe, for instance, the prevalence of OCA is as high as 1 in 1,000 ( Lund *et al.*, 1997). Alarmingly, OCA continues to be perceived with deep-rooted superstition in most parts of sub-Saharan Africa such that living with OCA is beset with severe psychological and social harms ( Hong *et al.*, 2006). These include being stigmatised, ostracised, physically abused and, not uncommonly, being gruesomely murdered for superstitious reasons ( Brilliant, 2015).

Clearly, therefore, the prospect of GGE in Africa promises unprecedented benefits including the radical physical healing of the highly prevalent genetic disorders; the emotional healing of affected individuals, families and communities; the elimination of associated stigmatisation; and the prevention of social harms, including death, to affected individuals such as those with OCA. Perhaps more urgently pertinent is the fact that Africa presents a unique opportunity for empirical research in human GGE because of its high prevalence of monogenic disorders. Evidently, as Wonkam (2023) rightly points out, human genomics research holds the future for the therapeutic management of sickle cell disease. Moreover, intensified human genomics research in Africa would yield an invaluable and pivotal contribution to the global elucidation of human genomics in general - such as in genome-wide association studies - because of the characteristic genomic diversity of African populations ( Pereira *et al.*, 2021). Conspicuously, however, there has been little to no attempt at comprehensively engaging the African public in discussions on the promises and concerns about HGE. Thus, the echoing key question remains as to how Africa should prudently embrace and govern the revolutionary biotechnology of HGE.

In this article, therefore, I lay the groundwork for the possible development of an appropriate ‘African framework for public engagement with HGE’ (AFPEHGE) and call upon all stakeholders to urgent synergistic action. First, I give an overview of the urgent need for authentic African public engagement with HGE. I then critically analyse the recently published World Health Organisation (WHO) framework for the governance of HGE, with a view to contextualising it to Africa. Lastly, I discuss some practical considerations for synergistic action and highlight WHO’s possible leadership role in the urgent development of an appropriate AFPEHGE.

## The urgent need for authentic African public engagement with HGE

Unlike the generally uncontroversial somatic cell gene editing, human GGE is beset with serious ethical concerns especially because of the significant risks of inadvertent and intentional misuse of its transgenerational heritability ( Gyngell *et al.*, 2017). Objections to the pursuit of human GGE include the unproven safety of the CRISPR method, unintended heritable off-target mutations, unknown future consequences of germline changes, the misrepresentation and lack of consent of future generations, the misuse of GGE for human enhancement rather than disease prevention and treatment, and the use of GGE for eugenics purposes ( Gyngell *et al.*, 2017). Many authors, thought leaders and expert organisations - mainly in the global North - have therefore called for due prudence in the pursuit of GGE ( Gyngell *et al.*, 2019). Calls for due prudence have become even more pronounced in the wake of the 2018 case of He Jiankui’s “CRISPR’d babies” ( Greely, 2019: 111). A global consensus on a moratorium on heritable HGE was emphatically reiterated at the recent Third International Summit on Human Genome Editing as highlighted in its 08 March 2023 Statement. Meanwhile, despite its high burden of genetic diseases, Africa is evidently lagging in articulating its position on human HGE. A 2020 global mapping survey, for instance, revealed no more than 5 African national policies with any clear position on human GGE ( Baylis *et al.*, 2020). There is an urgent need, therefore, for an appropriate framework for African public engagement with the revolutionary biotechnology of HGE. Such a framework would have to take into account Africa’s heterogeneity, commonalities, realities and complex value systems that would inform the prudent pursuit of HGE in general and GGE in particular.

Notably, African traditional thought cherishes the virtue of prudence as reflected in the many African wise sayings and proverbs. Among the Baganda, for instance, there is a prudential saying that ‘ *bwogendanga gyotomanyi omalanga kubuuliriza’*, which loosely translates that ‘whenever you travel to any unfamiliar place, you should always be cautious and first make the necessary enquiries about that place’ so as to avoid possible grave dangers. Similarly, the Swahilis rightly caution that ‘ *moto usioweza kuuzima usiwashe’*, meaning that one should never start a fire that one cannot control or extinguish. The key message here is that one must always be prudent and take the necessary precautions in any new venture to avoid, or at least minimise, any dangerous outcomes. Essentially, according to African traditional thought, human GGE poses unprecedented ethical concerns not only because of the evident scientific uncertainties about its transgenerationally heritable consequences, let alone its possible misuse for eugenics purposes, but also because of its apparent threat to the deep-rooted African communitarian worldview ( Kasenene, 2000; Mbiti, 1970; Tempels, 1959) and reverence for human life ( Rakotsoane & van Niekerk, 2017).

Prudence in African traditional thought demands, therefore, that Africa proactively engages in an authentic enquiry into the novel biotechnology of human GGE before it may embrace it for its potential benefits. Hence, there is an urgent need to develop and implement an appropriate African value-laden public engagement framework whereby researchers in HGE adequately address the views and concerns of the various publics in Africa. These publics and key stakeholders include patient groups, health care professionals, research ethics committees, academics, politicians, traditional leaders, religious leaders, media practitioners, youth groups, businesspeople, and marginalised groups, among others. It is vital to note that African public engagement with HGE is neither a tacit, or gravitational, endorsement of heritable HGE at its current level of advancement nor antithetical to its current international prohibition. Rather, authentic public engagement will clarify misconceptions and empower the public to deliberate and make value-laden decisions about the potential benefits and inherent harms, as well as the ethical, legal and social implications of GGE, so as to rightfully contribute to ethical policy formulation and governance ( Andorno *et al.*, 2020; de Miguel Beriain, 2021; Johnson, 2021). In other words, public engagement is precautionary, and a precautionary approach ( Martuzzi & Tickner, 2004) to GGE is quite evident in the recently published WHO framework for the governance of HGE.

## The timely WHO framework for governance of human genome editing

In July 2021, WHO published its guidance framework for the governance of HGE ( WHO, 2021a). This was a culmination of two years of intensive and extensive work by the WHO *Expert Advisory Committee on Developing Global Standards for Governance and Oversight of Human Genome Editing* (hereafter referred to as the WHO Expert Advisory Committee (WHO-EAC)), in the wake of the advent of CRISPR-Cas9. Drawing from best practices in the governance of emerging biotechnologies, the document defines governance “as including the norms, values and rules of the processes through which public affairs are managed so as to ensure transparency, participation, inclusivity and responsiveness” ( WHO, 2021a: x). Hence, good governance is necessarily a proactive, ongoing iterative process that thrives on public confidence. It is thus based on ethical values and principles and rooted in authentic public engagement ( WHO, 2021a).

In its governance framework for HGE, WHO outlines several key values and principles mainly pertaining to research integrity, responsibility, justice, and personal autonomy ( WHO, 2021b). It is worth noting that the WHO-EAC recognises the evident lack of harmonisation of these values and principles and calls upon the WHO Director-General to prioritise the development of an official WHO ethical framework ( WHO, 2021b). However, because it is specifically limited to public health interests ( WHO, 2021b), the WHO-EAC’s recommendation falls short of addressing the need for a comprehensive ethical framework that is rooted in societal values and principles. Essentially, a value refers to the transcendental notion of the good ( Lonergan, 1972). Thus, values are qualities that human beings consider to be good and important - such as physical, spiritual and social well-being; truthfulness; fairness; preservation of innocent human life; and ubuntu, just to mention a few. Societal values eventually generate societal norms, or generally acceptable and expected behaviour, for which ethical principles may be derived for value-laden action guidance. It is imperative, therefore, that ethical governance of HGE, and indeed of any novel biotechnology, reflects authentic societal values and principles.

The WHO governance framework for HGE is evidently content-thin on the human values at stake regarding the unprecedented advances in the biotechnology of HGE. For instance, while similar guidance documents on HGE - such as those of the United Nations Educational, Scientific and Cultural Organisation’s International Bioethics Committee (2015) and the European Commission’s Directorate-General for Research and Innovation (2021) - recognise and cite the dignity of the human person as the foundational and overarching human value at stake in HGE, there is not a single explicit mention of ‘human dignity’ in the WHO governance framework document. Admittedly, there may be implied reference to human dignity in the document’s mention of ‘respect for persons’ and ‘equal moral worth’ as being among the necessary values and principles for the good governance of HGE. It is also understandable that the concept of human dignity may sometimes be ambiguous and paradoxical ( Andorno, 2011; Bayertz, 1996). Its explicit exclusion in an international governance framework for HGE, however, risks alienating a significant part of the international public for whom human dignity is understood as the overarching principle of international biomedical law ( Andorno, 2013). Indisputably, human dignity is an ordinary human experience and the basis for human rights ( United Nations, 1948), and human rights are the juridical concretisation of human dignity ( Hailer & Ritschl, 1996).

As Spiegelberg (1971) rightly observes, the concept of human dignity is arguably the common denominator in our world of moral relativism. Indeed, the dignity of the human person is intrinsic and inalienable, simply arising from the essentially rational nature of the human being ( Lee & George, 2008; Ssebunnya, 2013). To contextualise this in African traditional thought, the concept of human dignity is encapsulated in the ethical value of ‘common humanness’ - variously referred to as ubuntu, *botho*, *utu,* etc., in many parts of Africa. Certainly, human dignity is enshrined in the African traditional cosmological worldview of harmonious existential vitality, which is characterised by some metaphysical “vital force” ( Kasenene, 2000: 349; Tempels, 1959: 21-22) and reverence for human life ( Rakotsoane & van Niekerk, 2017). Moreover, in African traditional ontology, human life refers not only to the living but also to “the living-dead” ( Mbiti, 1970: 107) as well as to future generations *.* Hence, in Africa, reference to human dignity becomes crucial to any authentic debate on HGE, particularly regarding the transgenerational heritability of GGE. Favourably, the WHO governance framework fundamentally endorses value-laden, public engagement with HGE ( WHO, 2021b). According to WHO, the issue of public engagement is central to the ethical governance of HGE particularly because HGE potentially affects the whole of humanity ( WHO, 2021b). Essentially, therefore, public engagement with HGE is a global existential priority – including in Africa, Africa’s evident resource-limited setting and competing priorities ( Andorno, 2022) notwithstanding. In fact, the fundamental issue is about, among others, 1) re-affirming and safeguarding the universal inalienable dignity of the human person ( Lanigan, 2008) in the advent of probable human GGE, 2) equity in human genomics research and healthcare ( Munung *et al.*, 2022), and 3) ethically harnessing the invaluable contribution - to global human genomics research and healthcare - of the unique African genomics diversity ( Pereira *et al.*, 2021). In other words, without authentic African public engagement with emerging innovative biotechnologies, such as HGE, there is a real danger of violation of the dignity of the human person, ethical imperialism, epistemic colonialism, inequity and predatory collaborations, as Pratt and de Vries (2023) rightly point out.

## Towards authentic African public engagement with human genome editing

Broadly conceived, public engagement is concerned with involving the public in the activities of setting the agenda, deliberation, and policy formulation for the ethical governance of a given intervention ( Rowe & Frewer, 2005). Hence, authentic public engagement must articulate fair and efficient mechanisms for three essential components, namely public communication, public consultation and public participation ( Rowe & Frewer, 2005). Characteristically, the flow of information in public communication is from the initiating expert(s) to the public. Conversely, the flow of information in public consultation is essentially the other way around. Then, in public participation, the two parties must genuinely contribute together, through dialogue, in policy decision-making. As Scheufele *et al.* (2021) aptly expound, there are at least seven essential goals for authentic public engagement with frontier science and technologies such as HGE. Thus, there are two essential goals with respect to public communication: 1) informative public education, and 2) correcting misconceptions and avoiding potential controversy (as happened with ‘genetically modified organisms’). There are also two essential goals with respect to public consultation: 1) democratic deliberation, and 2) inclusive representation, whereby all views, including those of minorities and the marginalised, are equally valued. Lastly, there are three essential goals with respect to public participation: 1) discerning, articulating and deliberating on the complex values at play to resolve any dilemmas, 2) fostering responsible innovation, and 3) consensual policy formulation for ethical governance. Given the unprecedented potential benefits and serious ethical concerns posed by the prospect of GGE, comprehensive public engagement with HGE (as outlined above) clearly becomes an indisputable imperative ( Adashi *et al.*, 2020), especially among marginalised publics and vulnerable populations ( WHO, 2021b) such as in Africa.

Indeed, the issue of African public engagement with HGE becomes compounded by the continent’s perennial resource constraints. Favourably, however, there are some incipient efforts towards African public engagement with emerging biotechnologies, especially in the area of genetic research. An outstanding example in this regard is the work done by the Human Heredity and Health (H3Africa) consortium. The H3Africa consortium was established in 2012 and, according to its strategic intent, aims at facilitating responsible research in genomics towards human flourishing in Africa. To this end, the consortium identified community engagement as a key ethical challenge to research in African genomics and, therefore, specifically developed a set of guidelines for its community engagement activities ( H3Africa Community Engagement Working Group [H3Africa CEWG], 2017; Tindana *et al.*, 2017). However, it is crucial to note the narrower scope of ‘engagement’ in community engagement as compared to public engagement. While community engagement focuses on specific communities directly involved or affected in a particular research context, public engagement is aimed at engaging all the various publics (directly involved or not) that may be concerned about a particular biotechnological intervention ( H3Africa CEWG, 2017). Nonetheless, given the diffuse boundaries and overlap of activities between the two ( H3Africa CEWG, 2017), the lessons learned from H3Africa’s experience of community engagement can richly inform the development of an appropriate AFPEHGE.

Furthermore, the current efforts by H3Africa and the African Society of Human Genetics, through the African Genomic Medicine Training Initiative (AGMT), are also greatly contributory and indeed commendable. The AGMT aims at training nurses, medical doctors and medical laboratory professionals in the essentials of African genomics ( Nembaware *et al.*, 2019). In addition to the science of genomic medicine, the AGMT course contains a vital module on the ethical, legal and social implications (ELSI) of genomics. Notably, however, the AGMT public communication initiative is also still limited in outreach and scope (largely focusing on healthcare professionals), and its future currently depends on continued availability of external funding. Nonetheless, it is plausible that such training may be extensible for other stakeholder publics - including academics, politicians, traditional leaders, religious leaders, media practitioners, youth groups, businesspeople, and marginalised groups, among others. As Gyngell *et al.* (2019) rightly observe, the responsible way to enable the public to make ethical decisions about emerging biotechnologies is to educate the public on the fundamentals of the relevant science and ethics. Also notable are the significant initiatives towards public consultation and participation, particularly with regard to genomic research and biobanking, albeit in the limited context of research-related community engagement ( de Vries *et al.*, 2017; Tindana *et al.*, 2019; Yakubu *et al.*, 2018). Evidently, the need for a comprehensive AFPEHGE remains acute and calls for prompt action.

## Next steps in developing an appropriate AFPEHGE

It emerges, therefore, that authentic African public engagement with HGE requires a concerted multi-disciplinary approach headlined by both genomics scientists and ELSI experts. Hence, I submit that the most critical next step towards developing an appropriate AFPEHGE is to establish a dedicated pan-African AFPEHGE working group (AFPEHGE-WG) of genomic scientists and ELSI experts. The latter should have the necessary research competencies in the ethical, legal, and social implications of emerging biotechnologies, particularly human genome editing. As already indicated above, the essential role of the genomic scientists is in engaging the public with the fundamentals of the science of HGE. The complementary task for the ELSI experts would necessarily entail the articulation of contemporary African thought on the fundamental concepts in the critical debate on HGE. These include such contentious concepts like human nature, human personhood, human dignity, human rights and the common good, among others. These universal fundamental concepts are habitually reflected in ordinary human experience, and their articulation is arguably essential in informing the debate on such an existential issue like human GGE. Indeed, these foundational concepts, properly contextualised, underpin the African value system by which the issue of HGE may be authentically evaluated in Africa.

It is vital to note that, despite the commonality of its postcolonial realities, aspirations and disease burden, Africa is not a homogeneous entity ( Ssebunnya, 2022). Hence, an appropriate AFPEHGE-WG would have to reflect the heterogeneous nature of African value systems. A pragmatic approach would be to adapt WHO’s Sub-Saharan African region and subdivide it into four constituent sub-regions namely, western, central, eastern and southern. Accordingly, the AFPEHGE-WG could recruit – on merit - at least one genomic scientist and one ELSI expert from each of the four sub-regions. Thus a representative pan-African AFPEHGE-WG would consist of at least eight subject experts who would also engender local capacity building. The resolute mission of a duly constituted AFPEHGE-WG, therefore, would be to spearhead the development of a credible value-laden AFPEHGE

In order to realise its strategic intent, the AFPEHGE-WG could adapt, for instance, the well tested ‘logic model’ that facilitates the planning and evaluation of complex public engagement interventions ( National Co-ordinating Centre for Public Engagement [NCCPE], n.d.; NCCPE, 2017). The AFPEHGE-WG’s task would then typically involve 1) a comprehensive and realistic assessment of the current situation, and scoping of the AFPEHGE, by way of a landscape analysis, 2) setting the aims to be achieved, 3) specifying the necessary inputs, 4) determining and designing the requisite activities to achieve the set aims, 5) specifying the outputs to be created, 6) indicating the resulting outcomes to guide ethical governance and policy formulation on HGE, 7) indicating the long-term impact of the AFPEHGE, 8) indicating the assumptions made in the designed approach, and 9) identifying and possibly addressing the external factors that could influence the project outcomes. Each of these features of the logic model of public engagement necessitates detailed and contextualised consideration by the AFPEHGE-WG. Favourably, there are a number of handy resources available for benchmarking, such as the Genomics Education (GenomeEd) Resources, of the National Human Genome Research Institute; the Personal Genomics Education (pgEd) Project programmes; the MESH of the Global Heath Network; and the Genome Editing Public Engagement Synergy, of the NCCPE.

Given the significant inequities and resource limitations in Africa, perhaps the most crucial consideration by the AFPEHGE-WG would be how to foster authentic public consultation. To avoid mere tokenism, authentic public consultation demands authentic democratic deliberation, as already noted above, which is essentially an inclusive method of decision-making that values the views and contributions of all members of a given public on a given policy issue ( Presidential Commission for the Study of Bioethical Issues, 2016). As many authors ( De Vries *et al.*, 2019; Dror *et al.*, 2007; Goold *et al.*, 2005; Gutmann & Wagner, 2017; Tomlinson *et al.*, 2018; and Fleck, 1994) have ably argued, effective democratic deliberation on novel biotechnologies and healthcare interventions is indispensable for ethical governance and policy formulation. Indeed, as Gusmano *et al.* (2021) rightly reiterate, democratic deliberation is vital not only to allay fears and clarify misconceptions (on the side of the public) but also for the researchers and policymakers to harness and learn from the sometimes-deep-seated wisdom of members of the public.

Furthermore, there is no doubt that the most critical challenge to the establishment and effective functioning of a competent AFPEHGE-WG is the securing of adequate funding, among other resources. Hence, establishing an effective AFPEHGE-WG calls for synergistic collaboration among relevant governmental institutions, non-governmental organisations, international development partners, and others. Arguably, the most pragmatic way would be to rally behind the WHO-EAC’s recommendation for a collaborative approach to public engagement with HGE ( WHO, 2021b). In particular, the WHO-EAC calls upon WHO to partner with other organisations and explore how to ensure proper inclusion of under-represented groups in the development of guidance on HGE and earmark the necessary funding for this vital undertaking ( WHO, 2021b). Hence, given WHO’s established mandate, infrastructure and compelling recommendations for the governance of HGE, it is conceivable that WHO could – and arguably should - rally the various stakeholders and Africa’s development partners, and champion the establishment of the AFPEHGE-WG.

## Conclusion

In this open letter, I have highlighted the urgent need for authentic African public engagement with HGE and WHO’s possible leadership role in the development of the requisite AFPEHGE. There is no doubt that Africa stands to exponentially benefit from the revolutionary CRISPR-Cas9 method of HGE. Moreover, as I have pointed out, Africa’s high prevalence of monogenic disorders presents a unique opportunity for empirical research in human GGE. Hence, given the serious ethical concerns regarding the issue of transgenerational heritability in GGE, Africa cannot afford to ignore the evident serious lag in engaging African publics with HGE. To re-iterate, authentic African public engagement with HGE would require the establishment of a competent AFPEHGE-WG capable of discerning the complex African value systems, realities, needs and concerns about HGE. Due to Africa’s manifest resource constraints, especially in securing adequate funding, there is an urgent need for synergistic collaboration among all stakeholders. Favourably, this call to synergistic action aligns with WHO’s timely recommendations for the ethical governance of HGE. WHO could therefore champion the commissioning of an AFPEHGE-WG to spearhead authentic African public engagement with HGE.

## Data Availability

No data are associated with this article.
