# Supplementary material for: Towards an appropriate African framework for public engagement with human genome editing: a call to synergistic action
Source: Wellcome Open Res. 2023 May 12;7:302. Originally published 2022 Dec 12. [Version 2] doi: 10.12688/wellcomeopenres.18579.2 (PMC10359742; doi:10.12688/wellcomeopenres.18579.2)
Supplement: Peer review history

# Peer review: Reviewer response for version 2

**DOI:** 10.21956/wellcomeopenres.21552.r57343

**Published:** 2023-07-20

**Authors:** Roberto Andorno

**Affiliations:** 1 Faculty of Law and Institute of Biomedical Ethics and History of Medicine, University of Zurich, Zürich, Switzerland

The author has taken into account most of the remarks I made in my first review and has significantly improved the quality of the manuscript. The current version is more nuanced and mentions more explicitly the serious ethical issues posed by human germline gene editing (HGGE).

 However, I still have one remark: the author has maintained a problematic statement in the abstract and in the Introduction saying that human germline gene editing (HGGE) is “relatively safe”. This is inaccurate, as I have pointed out in my previous review, and needs imperatively to be corrected. All major scientific academies that have dealt with HGGE agree that this procedure is not safe to be used in human reproduction (not even “relatively safe”). This is precisely why all of them call for a moratorium in this area. The only paper cited in support of that problematic statement is the one by Kofler and Kraschel (2018). But that paper nowhere says that HGGE is “safe”. Therefore, I suggest the author to replace that statement with something more nuanced like, for instance: “this technique holds great promise for the prevention of monogenic disorders” or that it “could potentially be used to prevent monogenic disorders”.

Does the article adequately reference differing views and opinions?

No

Are all factual statements correct, and are statements and arguments made adequately supported by citations?

No

Is the Open Letter written in accessible language?

Yes

Where applicable, are recommendations and next steps explained clearly for others to follow?

Partly

Is the rationale for the Open Letter provided in sufficient detail?

No

Reviewer Expertise:

Bioethics; Human Rights; Medical Law.

I confirm that I have read this submission and believe that I have an appropriate level of expertise to confirm that it is of an acceptable scientific standard, however I have significant reservations, as outlined above.

# Peer review: Reviewer response for version 2

**DOI:** 10.21956/wellcomeopenres.21552.r57342

**Published:** 2023-05-22

**Authors:** Francis C. L. Rakotsoane

**Affiliations:** 1 Centre for Medical Ethics and Law, Department of Medicine, Faculty of Medicine and Health Sciences, Stellenbosch University, Stellenbosch, South Africa

The author has adequately addressed the concerns that I had raised on the unrevised version of the article. I, therefore, recommend the indexing of the article.

Does the article adequately reference differing views and opinions?

Yes

Are all factual statements correct, and are statements and arguments made adequately supported by citations?

Yes

Is the Open Letter written in accessible language?

Yes

Where applicable, are recommendations and next steps explained clearly for others to follow?

Yes

Is the rationale for the Open Letter provided in sufficient detail?

Yes

Reviewer Expertise:

I am a biomedical ethicist with a special interest in biomedical research topics.

I confirm that I have read this submission and believe that I have an appropriate level of expertise to confirm that it is of an acceptable scientific standard.

# Peer review: Reviewer response for version 1

**DOI:** 10.21956/wellcomeopenres.20601.r54673

**Published:** 2023-03-28

**Authors:** Dorcas M. Kamuya

**Affiliations:** 1 Health Systems and Research Ethics, KEMRI-Wellcome Trust Research Programme, Kilifi, Kenya; 2 Nuffield Department of Medicine, University of Oxford, Oxford, UK

This open letter is timely on an area that needs urgent attention. However, it needs to be revised significantly. There is uncritical use of words and concepts for example what is African? Africa is quite diverse and heterogenous in so many ways; even in any one country there are multiple ethnic communities; yet throughout the author describes Africa as one homogenous community bounded by some common values (even when he/she acknowledges that is not the case, rather than engaging with this multiplicity and the implications for the proposed engagement framework). Similarly, what is authentic - a word that is used in many places throughout the letter. There also seems to be some assumptions that 'anything African is authentic" and there is no critique or reflection about this particular gaze. Some of the words and expressions lean towards journalist expressions rather presenting arguments that invite the readers to make similar conclusions as the author. 

 There is incredible scholarly work on community and public engagement in health research generally and on genetic research and biobanking specifically, including from LMIC. The author seems not to have engaged with this literature well. While I agree that engagement on Human Genome Editing is lagging and therefore support the focus on this aspect, still there is a lot that can be learnt from earlier mentioned engagement efforts. For example, many engagement researchers and practitioners see information and consultation as a somewhat low level of engagement compared to engagement that fosters relationship-building partnerships between researchers and communities. In the latter, research is co-created and co-produced with communities and publics. This is a point that seems to be underplayed in the letter. 

 In addition to addressing the above issues, it is not clear how the proposed public engagement is different from already widely document engagement approaches and frameworks, including in GCP. A recent realist review of engagement (hosted in the MESH website of the Global Heath Network site) would be worth looking at. I would have liked to see a critique of the proposed 'well tested logic model' which is proposed, and an articulation of the values that are suggested and how these might play out in engagement on HGE.

 Finally, while there some flaws in this letter as stated above, the call it makes is timely, and I would recommend a revision that takes account of the issues raised.

Does the article adequately reference differing views and opinions?

No

Are all factual statements correct, and are statements and arguments made adequately supported by citations?

No

Is the Open Letter written in accessible language?

Yes

Where applicable, are recommendations and next steps explained clearly for others to follow?

Partly

Is the rationale for the Open Letter provided in sufficient detail?

Yes

Reviewer Expertise:

Engagement, Ethics, social science

I confirm that I have read this submission and believe that I have an appropriate level of expertise to confirm that it is of an acceptable scientific standard, however I have significant reservations, as outlined above.

# Response

**Published:** 2023-05-01

**Authors:** Gerald Michael Ssebunnya

**Affiliations:** Africa Institute for Human Dignity, Botswana

I wish to thank the reviewer for the insightful and constructive comments. Accordingly, in the revised version (RV) of my open letter,

1. I have highlighted the fact that Africa is not a homogeneous entity and, hence, the need for a representative pan-African AFPEHGE-WG. I have pragmatically aligned the AFPEHGE to the WHO African Region, thus focusing on Sub-Saharan Africa (RV, paragraph 2 of the section on the “Next steps in developing an appropriate AFPEHGE”).
2. I have deleted the word ‘authentic’ where it appeared rather redundant. I have retained it, in its ordinary meaning, where it is needed to denote the genuineness of intent, planning and execution of public engagement with human genome editing, in Africa - as opposed to mere tokenism, ethical imperialism, epistemic colonialism, or predatory collaboration (Pratt & de Vries, 2023). I have underscored this point in the RV (paragraph 5 of the section on “The timely WHO framework for governance of human genome editing”).
3. The letter highlights the vital distinction between community engagement and public engagement, particularly citing the commendable work done in Africa by the H3Africa Consortium (paragraph 2 of the section on “Towards authentic African public engagement with human genome editing”).
4. The letter highlights the three essential components of public engagement, namely public communication, public consultation and public participation (Rowe & Frewer, 2005); and explains their seven essential goals with regard to frontier biotechnologies as expounded by Scheufele et al (2021) (paragraph 1 of the section on “Towards authentic African public engagement with human genome editing”). The ‘logic model’ of public engagement is cited simply as a relevant example that could be adapted as determined by the AFPEHGE-WG (and I have rephrased accordingly for clarity).  Hence, I have deferred the contextualised critique of the ‘logic model’ - and others - to the AFPEHGE-WG, limiting the scope of my article to the core argument that there is an urgent need for an appropriate AFPEHGE and AFPEHGE-WG.
5. The MESH website of the Global Heath Network is certainly a handy resource and I have added it to the list of those I have cited (RV, paragraph 4 of the section on the “Next steps in developing an appropriate AFPEHGE”). 

**References**

1. Pratt, B., & de Vries, J. (2023). Where is knowledge from the global South? An account of epistemic justice for a global bioethics. *Journal of medical ethics*, 49(5), 325–334. https://doi.org/10.1136/jme-2022-108291
2. Rowe, G. & Frewer, L.J. (2005). A Typology of Public Engagement Mechanisms. *Science, Technology, & Human Values*, 30(2), 251-290. https://doi.org/10.1177/0162243904271724 
3. Scheufele, D. A., Krause, N. M., Freiling, I., & Brossard, D. (2021). What we know about effective public engagement on CRISPR and beyond. *Proceedings of the National Academy of Sciences of the United States of America*, 118(22), e2004835117. https://doi.org/10.1073/pnas.2004835117

# Peer review: Reviewer response for version 1

**DOI:** 10.21956/wellcomeopenres.20601.r55340

**Published:** 2023-03-08

**Authors:** Francis C. L. Rakotsoane

**Affiliations:** 1 Centre for Medical Ethics and Law, Department of Medicine, Faculty of Medicine and Health Sciences, Stellenbosch University, Stellenbosch, South Africa

The article is titled "Towards an Appropriate African Framework for Public Engagement with Human Genome Editing: A Call to Synergistic Action". In the article, the author identifies the non-existence of this kind of framework at present as a major stumbling block to authentic democratic deliberation when engaging the public on issues of human genomic studies in Africa. Having outlined the problems and challenges, the author then proposes an establishment of what he calls a dedicated African framework for public engagement with human genome editing working group (AFPEHGE-WG) that should consist of both genomic scientists and Pan-African ELSI experts whose task should be to help in determining the ethical, legal, and social implication of genomic studies in Africa. The author then goes on to give a nine-pointed task for the proposed working group.

 The author has not, however, spelled out the kind of experts that should constitute his proposed ELSI experts group. It is important to have this clearly spelled out so that people do not have to scratch their heads as to who they should include in the group. It would be ideal to have such a group constituted in the same way for quality assurance purposes if the proposed framework is to serve as a generic framework for a large part of the African Continent as envisaged by the author (I suppose). It would have been more helpful if the author had also indicated the number of ELSI experts that have to be in the proposed group.

 Another important factor that the article’s author has to take into consideration is the fact that Africa is not a homogeneous continent. It consists of regions and sub-regions whose peoples' worldviews are, sometimes, diametrically opposed to each other. Compare the Northern African region with Southern Africa, for example. It is, therefore, advisable to indicate clearly what part of Africa the proposed “African framework” is meant to cover. Is it for the whole continent or just a part of the continent? Is it intended for the whole of sub-Saharan Africa or just for the Southern African region?

 The framework that the author seeks to have in place will undoubtedly be a vital tool for facilitating research not only on human genome editing but also for encouraging collaborations among African researchers, as well as with scientists outside of Africa as advocated for in one of the Human Heredity and Health in Africa (H3Africa) Consortium objectives. The existence of a framework of this nature will help in having the said collaborations take place unhampered by communities’/participants’ refusal to fully cooperate on account of feeling not fully or adequately engaged, as is currently the case.

 Please note that the name Rakotsoane referred to on pages 5 and 7 of the letter is misspelled. There should be an "o" in front of a "k" in Rakotsoane. It is Rak **o**tsoane not Rak **a**tsoane.

 On the basis of the foregoing remarks, I approve the article with reservations (the article requires further revisions before it can be considered to be fully scientifically valid).

Does the article adequately reference differing views and opinions?

Yes

Are all factual statements correct, and are statements and arguments made adequately supported by citations?

Yes

Is the Open Letter written in accessible language?

Yes

Where applicable, are recommendations and next steps explained clearly for others to follow?

Yes

Is the rationale for the Open Letter provided in sufficient detail?

Yes

Reviewer Expertise:

I am a biomedical ethicist with a special interest in biomedical research topics.

I confirm that I have read this submission and believe that I have an appropriate level of expertise to confirm that it is of an acceptable scientific standard, however I have significant reservations, as outlined above.

# Response

**Published:** 2023-05-01

**Authors:** Gerald Michael Ssebunnya

**Affiliations:** Africa Institute for Human Dignity, Botswana

I wish to thank the reviewer for the insightful and constructive comments. Accordingly, in the revised version (RV) of my open letter,

1. I have spelled out the kind and number of ELSI experts required for the AFPEHGE-WG (RV, paragraph 2 of the section on the *“Next steps in developing an appropriate AFPEHGE*”).
2. I have highlighted the fact that Africa is not a homogeneous entity and, hence, the need for a representative pan-African AFPEHGE-WG - pragmatically aligned to the WHO African Region, thus focusing on Sub-Saharan Africa (RV, paragraph 2 of the section on the “Next steps in developing an appropriate AFPEHGE”).
3. I have corrected the spelling of the name in the citation to Rakotsoane. My apologies for the misspelling in the initial version.

# Peer review: Reviewer response for version 1

**DOI:** 10.21956/wellcomeopenres.20601.r53821

**Published:** 2023-02-03

**Authors:** Roberto Andorno

**Affiliations:** 1 Faculty of Law and Institute of Biomedical Ethics and History of Medicine, University of Zurich, Zürich, Switzerland

The Open Letter by Gerald M. Ssebunnya emphasizes the need to promote public engagement around human germline gene editing (HGGE) from the perspective of African values. This statement is in conformity with the various recommendations made by experts and scientific bodies over the past few years (1). The reason for this is clear: the decision about whether, and if so, under what conditions we have the right to alter the genome of future children cannot be made just by scientists. On the contrary, we all as human beings, should have a say in how to deal with this issue as we all are stakeholders in this matter.

 Therefore, the Letter is right in stressing the need to encourage public deliberation on HGGE, also in Africa. Unfortunately, the piece has some important shortcomings:

1. It is virtually silent about the serious ethical objections that have been leveled against the creation of genetically modified children and the irreversible harm that may result from introducing genetic changes that would be inherited by future generations. While the Letter just mentions in passing the “serious ethical concerns” about HGGE (without specifying them), it takes an excessively assertive and over-optimistic (if not naïve) view of the “unprecedented benefits” (sic) that will result from implementing HGGE in Africa. In this regard, a more balanced and cautious view on the potential risks and benefits of HGGE would be desirable.
2. Some statements are not only unbalanced but also inaccurate. For instance, when it is said that the technique is “relatively safe” (Introduction). Even the most enthusiastic supporters of the use of HGGE in the future admit that the technique involves today serious risks, especially in terms of off-target effects on the children whose genomes will be engineered. For this reason, they consider that it would be irresponsible to proceed with clinical applications at this time (see, for instance, the reports and statements of the US National Academies of Science and Medicine from the past few years).
3. The author seems to be unaware of the worldwide legal consensus against HGGE. Legal instruments from intergovernmental organizations, such as UNESCO, explicitly condemn the alteration of the human germline as being “contrary to human dignity” (see art. 24 of the 1997 Universal Declaration on the Human Genome and Human Rights that was also approved by most African countries). In addition, at the level of domestic legislation, no less than 75 countries prohibit the use of genetically modified in vitro embryos to initiate a pregnancy (2). The legal context in which the global debate on HGGE takes place has to be mentioned, at least briefly.
4. There is a fundamental question that is strangely absent from the Letter: Is the creation of genetically-edited children really an urgent public health priority for Africa? How would this be compatible with the high levels of poverty and the difficulties to have access to basic healthcare services that characterize most African countries?

Does the article adequately reference differing views and opinions?

No

Are all factual statements correct, and are statements and arguments made adequately supported by citations?

No

Is the Open Letter written in accessible language?

Yes

Where applicable, are recommendations and next steps explained clearly for others to follow?

Partly

Is the rationale for the Open Letter provided in sufficient detail?

No

Reviewer Expertise:

Bioethics; Human Rights; Medical Law.

I confirm that I have read this submission and believe that I have an appropriate level of expertise to state that I do not consider it to be of an acceptable scientific standard, for reasons outlined above.

## References

[1] : Geneva Statement on Heritable Human Genome Editing: The Need for Course Correction. *Trends Biotechnol* .2020;38(4) : 10.1016/j.tibtech.2019.12.022 351-354 10.1016/j.tibtech.2019.12.022 32014274

[2] : Human Germ Line and Heritable Genome Editing: The Global Policy Landscape. *CRISPR J* .2020;3(5) : 10.1089/crispr.2020.0082 365-377 10.1089/crispr.2020.0082 33095042

# Response

**Published:** 2023-05-01

**Authors:** Gerald Michael Ssebunnya

**Affiliations:** Africa Institute for Human Dignity, Botswana

I wish to thank the reviewer for the very helpful comments and objections, which I have taken into account in the revised version (RV) of my open letter:

1. I have specified the serious ethical concerns and current objections to the pursuit of human GGE (RV, paragraph 1 of the section on “ *The urgent need for authentic African public engagement with HGE*”). I have also highlighted, as an example, the realistic unprecedented potential benefit of human GGE in the therapeutic management of sickle cell disease, as Wonkam (2023) ably argues (RV, paragraph 2 of the “Introduction”). 
2. I have rephrased the point on the relative safety of the CRISPR method for clarity (RV, paragraph 1 of the “Introduction”), making it consistent with CRISPR’s rapidly evolving safety profile (Henderson, 2023).
3. It is worth noting that, in line with the *“rapid advancements in genetics and genomics*” (International Bioethics Committee, 2015: 1), the global consensus highlighted in the 1997 *Universal Declaration on the Human Genome and Human Rights* was actually updated by UNESCO’s International Bioethics Committee in its 2015 *Report of the IBC on Updating Its Reflection on the Human Genome and Human Rights*, which I have already duly cited in the initial version of my article (paragraph 3 of the section on “ *The timely WHO framework for governance of human genome editing*”). In the RV (paragraph 1 of the section on  “ *The urgent need for authentic African public engagement with HGE*”), I have also indicated that the current “ *global consensus on a moratorium on heritable HGE was emphatically reiterated at the recent Third International Summit on Human Genome Editing as highlighted in its 08 March 2023 Statement*”. 
4. In my view, the fundamental issue is not whether  “ *the creation of genetically-edited children [is] really an urgent public health priority for Africa … [or]  [h]ow [this] would … be compatible with the high levels of poverty and the difficulties to have access to basic healthcare services that characterize most African countries*” (Andorno, 2022).  Rather, Africa’s resource-limited setting and competing priorities notwithstanding, and as I have highlighted in the RV (paragraph 5 of the section on “ *The timely WHO framework for governance of human genome editing*”), 
*"the fundamental issue is about, among others, 1) re-affirming and safeguarding the universal inalienable dignity of the human person (Lanigan, 2008) in the advent of probable human GGE, 2) equity in human genomics research and healthcare (Munung et al, 2022), and 3) ethically harnessing the invaluable contribution - to global human genomics research and healthcare - of the unique African genomics diversity (Pereira et al, 2021). In other words, without authentic African public engagement with emerging innovative biotechnologies, such as HGE, there is a real danger of violation of the dignity of the human person, ethical imperialism, epistemic colonialism, inequity and predatory collaborations, as Pratt and de Vries (2023) rightly point out".*
  I have also highlighted the fact that 
*"African public engagement with HGE is neither a tacit, or gravitational, endorsement of heritable HGE at its current level of advancement nor antithetical to its current international prohibition. Rather, authentic public engagement will clarify misconceptions and empower the public to deliberate and make value-laden decisions about the potential benefits and inherent harms, as well as the ethical, legal and social implications of GGE, so as to rightfully contribute to ethical policy formulation and governance (Andorno et al, 2020; Johnson, 2021; de Miguel Beriain, 2021)" (RV, paragraph 3 of the section on “The urgent need for authentic African public engagement with HGE”). *
5. It is my hope that the reviewer will reconsider and approve this timely call for synergistic action towards an appropriate African framework for public engagement with human genome editing.

**References**

1. Andorno R. (2022). Peer Review Report For: Towards an appropriate African framework for public engagement with human genome editing: a call to synergistic action [version 1; peer review: 2 approved with reservations, 1 not approved]. *Wellcome Open Res,* 7:302 https://doi.org/10.21956/wellcomeopenres.20601.r53821 
2. Andorno, R., Baylis, F., Darnovsky, M., Dickenson, D., Haker, H., Hasson, K., Lowthorp, L., Annas, G. J., Bourgain, C., Drabiak, K., Graumann, S., Grüber, K., Kaiser, M., King, D., Kollek, R., MacKellar, C., Nie, J. B., Obasogie, O. K., Tyebally Fang, M., Werner-Felmayer, G., … Zuscinova, J. (2020). Geneva Statement on Heritable Human Genome Editing: The Need for Course Correction. *Trends in biotechnology,* 38(4), 351–354. https://doi.org/10.1016/j.tibtech.2019.12.022
3. de Miguel Beriain, Í. (2021). The Geneva Statement on Heritable Human Genome Editing: A Criticism. *Trends in biotechnology*, 39(3), 219–220. https://doi.org/10.1016/j.tibtech.2020.11.004 
4. Henderson, H. (2023, March 29). CRISPR Clinical Trials: A 2023 Update. *Innovative Genomics Institute*. https://innovativegenomics.org/news/crispr-clinical-trials-2023/ 
5. International Bioethics Committee. (2015). *Report of the IBC on Updating Its Reflection on the Human Genome and Human Rights. *United Nations Educational, Scientific and Cultural Organisation. https://unesdoc.unesco.org/ark:/48223/pf0000233258
6. Johnson T. (2021). In Defense of Heritable Human Genome Editing: On the Geneva Statement by Andorno et al. *Trends in biotechnology*, 39(3), 218–219. https://doi.org/10.1016/j.tibtech.2020.11.002 
7. Lanigan, B.T. (Ed.). (2008). *Human Dignity and Bioethics*. Nova Science Publishers, Inc.
8. Munung, N. S., de Vries, J., & Pratt, B. (2022). Towards equitable genomics governance in Africa: Guiding principles from theories of global health governance and the African moral theory of Ubuntu. *Bioethics*, 36(4), 411–422. https://doi.org/10.1111/bioe.12995
9. Pereira, L., Mutesa, L., Tindana, P., & Ramsay, M. (2021). African genetic diversity and adaptation inform a precision medicine agenda. *Nature reviews. Genetics*, 22(5), 284–306. https://doi.org/10.1038/s41576-020-00306-8
10. Pratt, B., & de Vries, J. (2023). Where is knowledge from the global South? An account of epistemic justice for a global bioethics. *Journal of medical ethics*, 49(5), 325–334. https://doi.org/10.1136/jme-2022-108291
11. Wonkam A. (2023). The future of sickle cell disease therapeutics rests in genomics. *Disease models & mechanisms*, 16(2), dmm049765. https://doi.org/10.1242/dmm.049765
